# A Patient-Centered Primary Care Practice Approach Using Evidence-Based Quality Improvement: Rationale, Methods, and Early Assessment of Implementation

**DOI:** 10.1007/s11606-013-2703-y

**Published:** 2014-04-09

**Authors:** Lisa V. Rubenstein, Susan E. Stockdale, Negar Sapir, Lisa Altman, Timothy Dresselhaus, Susanne Salem-Schatz, Susan Vivell, John Ovretveit, Alison B. Hamilton, Elizabeth M. Yano

**Affiliations:** 1 VA HSR&D Center for the Study of Healthcare Innovation, Implementation, and Policy, VA Greater Los Angeles Healthcare System, VA Greater Los Angeles, 16111 Plummer Street, North Hills, CA 91343 USA; 2 David Geffen School of Medicine, University of California, Los Angeles, Los Angeles, CA USA; 3 RAND Corporation, Santa Monica, CA USA; 4 Department of Medicine, VA Greater Los Angeles Healthcare System, Los Angeles, CA USA; 5 VA San Diego Healthcare System, San Diego, CA USA; 6 Department of Medicine, University of California, San Diego, San Diego, CA USA; 7 Health Care Quality Initiatives, Newton, MA USA; 8 The Karolinska Institutet, Stockholm, Sweden; 9 Department of Health Policy and Management, UCLA Fielding School of Public Health, Los Angeles, CA USA; 10 VA QUERI Center for Implementation Practice and Research Support, VA Greater Los Angeles Healthcare System, Sepulveda, Los Angeles, CA USA

**Keywords:** quality improvement, primary care, patient-centered medical home, logic model, interdisciplinary leadership

## Abstract

**BACKGROUND:**

Healthcare systems and their primary care practices are redesigning to achieve goals identified in Patient-Centered Medical Home (PCMH) models such as Veterans Affairs (VA)’s Patient Aligned Care Teams (PACT). Implementation of these models, however, requires major transformation. Evidence-Based Quality Improvement (EBQI) is a multi-level approach for supporting organizational change and innovation spread.

**OBJECTIVE:**

To describe EBQI as an approach for promoting VA’s PACT and to assess initial implementation of planned EBQI elements.

**DESIGN:**

Descriptive.

**PARTICIPANTS:**

Regional and local interdisciplinary clinical leaders, patient representatives, Quality Council Coordinators, practicing primary care clinicians and staff, and researchers from six demonstration site practices in three local healthcare systems in one VA region.

**INTERVENTION:**

EBQI promotes bottom-up local innovation and spread within top-down organizational priorities. EBQI innovations are supported by a research-clinical partnership, use continuous quality improvement methods, and are developed in regional demonstration sites.

**APPROACH:**

We developed a logic model for EBQI for PACT (EBQI-PACT) with inputs, outputs, and expected outcomes. We describe implementation of logic model outputs over 18 months, using qualitative data from 84 key stakeholders (104 interviews from two waves) and review of study documents.

**RESULTS:**

Nearly all implementation elements of the EBQI-PACT logic model were fully or partially implemented. Elements not fully achieved included patient engagement in Quality Councils (4/6) and consistent local primary care practice interdisciplinary leadership (4/6). Fourteen of 15 regionally approved innovation projects have been completed, three have undergone initial spread, five are prepared to spread, and two have completed toolkits that have been pretested in two to three sites and are now ready for external spread.

**DISCUSSION:**

EBQI-PACT has been feasible to implement in three participating healthcare systems in one VA region. Further development of methods for engaging patients in care design and for promoting interdisciplinary leadership is needed.

**Electronic supplementary material:**

The online version of this article (doi:10.1007/s11606-013-2703-y) contains supplementary material, which is available to authorized users.

## INTRODUCTION

The Patient-Centered Medical Home (PCMH) is a broadly endorsed set of general principles for aligning care delivered in primary care (PC) settings with the needs and preferences of the patient populations served. The model, however, has proven challenging to implement, even in highly motivated practices.^1^ Prior work shows that the PC practices most successful in achieving PCMH goals have an internal capability for organizational learning and development.^2^ Promoting front line quality improvement skills and innovation in PC settings within the context of PCMH goals has, in turn, the potential to enhance ongoing organizational learning. There are few empirical studies, however, testing systematic methods for promoting a learning and improvement culture for PCMH.

The Veterans Affairs (VA) system used PCMH principles as the basis for its patient-centered care model, termed “Patient Aligned Care Teams” (PACT). Nationally mandated PACT implementation began in 2010.^3^ Simultaneously, VA established demonstration projects to support PACT implementation through innovation and evaluation.^3^ We report here on the logic model, methods, and early implementation of a demonstration project to test Evidence-Based Quality Improvement (EBQI)^4^^–^ 7 as a method for developing and spreading a culture of quality improvement in primary care settings within one large VA region.

Centrally driven initiatives can founder on challenges faced by local sites in trying to implement them.^8^ Achieving mandated PACT goals such as interdisciplinary continuity of care for patient panels, new scheduling methods, and improved care transitions requires substantial local redesign.^8^ Prior literature on non-VA PCMH shows that model implementation is transformative, requiring multi-dimensional changes^9^ that continuously adapt to local context.^10^ We thus expected that implementation of PACT would necessitate an ongoing local quality improvement (QI) process, in addition to top-down mandates and education.^11^ Local QI innovation, however, can be both idiosyncratic^12^^,^ 13 and expensive.^14^

EBQI, tested over the past two decades within and outside VA,^4^^–^ 6 is a multilevel approach that has the potential to focus and empower local QI innovation. The approach integrates system-level and region-level improvement priorities^15^ with locally driven, bottom-up, evidence-based problem-solving that is supported by embedded health services researchers. By doing so, EBQI aims to promote both evidence-enriched local innovation and a culture in which local problem-solving is the norm.^16^^,^ 17

We adapted EBQI based on the particulars of the PCMH model, and termed our resulting approach EBQI-PACT. Compared to prior EBQI,^4^^–^ 7 EBQI-PACT more specifically emphasizes interdisciplinary practice leadership, patients as stakeholders, and methods for spreading innovations. This paper uses QI and qualitative methods to describe 1) the evidence, theories, and context that shaped the EBQI-PACT intervention; 2) the resulting project logic model; and 3) early implementation of program components across six demonstration primary care practices in one VA region (June 2010 to December 2012).

## METHODS

### Setting

The VA has a national central office, 21 administrative regions (termed Veterans Integrated Service Networks or VISNs), and local healthcare systems (HCSs, often called medical centers) within each region that in turn directly administer local primary care (PC) practices of varying size and complexity. The demonstration project reported here is the “Veterans Assessment and Improvement Laboratory for Patient-Centered Care” (VAIL-PCC, or VAIL), funded by the VA Office of Patient Care Services. VAIL is based within VISN 22, a region with five local HCSs, three of which participate in VAIL and administer 23 of the 35 total PC practices in the region. Each participating system chose one demonstration practice in year one (2010) and one additional practice in year two (2011), for a total of six. VAIL specified that any VA-staffed PC practice serving 7,000–15,000 veterans and willing to participate in QI was eligible.

**Figure 1:**
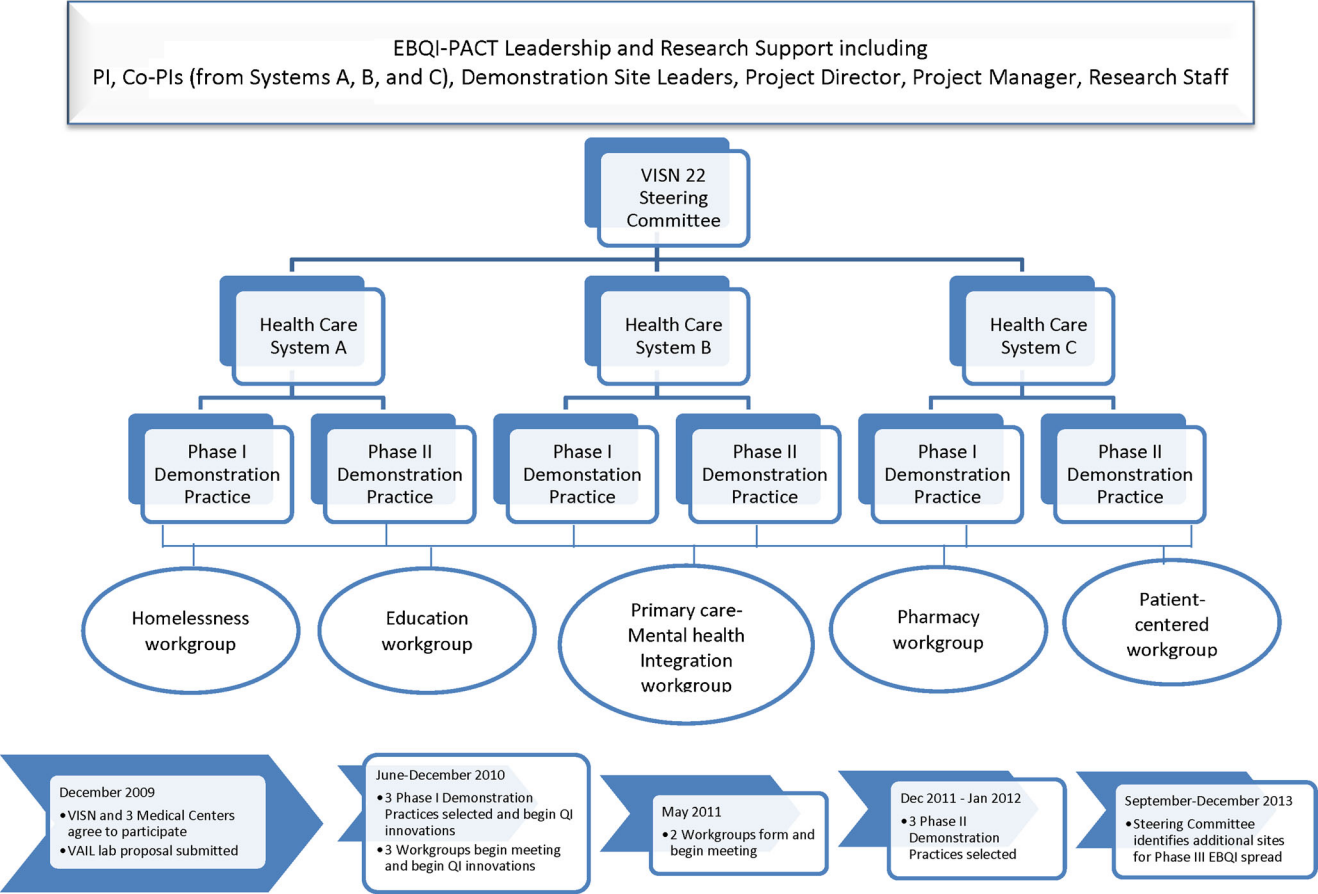
Organizational chart and time line for VAIL EBQI-PACT intervention.

EBQI-PACT is the core intervention sponsored by VAIL. VAIL additionally supports a separate four-year summative evaluation led by an organizational epidemiologist (EY) not yet concluded and not reported here. Figure 1 overviews the EBQI-PACT intervention and time line.

### Participants

VAIL involves over 200 direct participants in its activities, including regional, HCS, and local clinical leaders; patient representatives; Quality Council Coordinators; practicing PC clinicians and staff; and researchers. The researchers supporting EBQI–PACT intervention activities at least part-time include a PC MD (LR), a PhD sociologist (SES), a project manager (NS), a VA information systems specialist (SV), a programmer, a communications lead, two evidence review leads, two human subjects leads who are also survey experts, and several staff.

### Theoretical Basis for EBQI-PACT Intervention Features

The research team, with input from PC leads from each participating health system and a redesign expert based at regional headquarters, reviewed evidence from literature searches^18^ and consulted with experts to develop the rationale for the EBQI-PACT intervention in 2009–2010, prior to project funding. In Table 1, Column A shows challenges identified through PCMH literature. Column B shows relevant theories underlying EBQI. Column C shows related key EBQI-PACT features we expected to implement based on challenges and theories.

**Table 1:** Theoretical Basis for EBQI-PACT Intervention Features

A. Primary care improvement challenges	B. Relevant theoretical basis for intervention development	C. Relevant intervention features
“Becoming a PCMH requires transformation, not incremental change”^9^	• Leadership commitment^29^^–^ 31	• Link top-down leadership mandates with bottom-up clinic-level transformation
	• QI expert panel approaches^15^	
Transformation involves major shifts in roles and mental models within primary care and among its stakeholders^32^	• Primary care practice autonomy (EY)	• Local interdisciplinary leadership and QI support structures
	• Interdisciplinary boundary spanning coordination^33^	
“The technology needed for the PCMH is not plug and play”^9^	• CQI culture^34^^–^ 36	• QI coaching
	• Improvement design theory (The Chronic Care Model)^25^^,^ 26	• Formal innovations development using CQI methods
	• CQI diagnostic and analytic methods^37^	
	• Researcher/clinical partnership^38^	• Researcher/clinical partnership QI to promote evidence-informed locally initiated innovations
	• Knowledge transfer^39^^,^ 40	• Engagement of regional experts to support innovation in key topic areas
	• Program evaluation^41^^–^ 43	• Formative evaluation
Motivations of key stakeholders for understanding and guiding practice change influences PCMH success^44^	• Complex adaptive systems^1^	• Multilevel interdisciplinary and patient engagement
	• Multiple stakeholder engagement^45^	
	• Patients as stakeholders^46^	
	• Social marketing^47^	• Communications targeting
The complexity of PCMH implementation warrants efforts to address local contexts through spread of successful approaches^29^	• Diffusion of innovations^11^^,^ 48	• Demonstration site focus • Spread tools and process
	• Improving interdependencies for promoting spread^45^	
	• Frontline QI attitudes regarding support spread and adoption of innovations^49^^,^ 50	

Based on the theories and features, interviews with intervention and evaluation leaders (EY, LR, SS), and group discussions with the full research team, one of the authors (SSS) developed a first draft logic model (Appendix 1, available online). The final logic model, completed after field testing, reflects agreement among the authors on an abbreviated version.

### Evaluation of Implementation of EBQI-PACT

We used data from 104 qualitative key stakeholder interviews and from study records to assess fidelity to intended EBQI-PACT logic model elements between June 2010 and December 2012. The 30–60 min interviews were conducted in two waves with 84 VAIL participants (first wave interviews October 2011–July 2012, second wave interviews September 2012–March 2013). Trained qualitative researchers conducted the interviews, which covered predefined domains, and were audio-recorded and professionally transcribed. Approximately 80 % of invited stakeholders participated (first wave: 58/75; second wave: 46/54). The number of key stakeholders interviewed per HCS varied based on HCS size and leadership structure (ranging from 11 at the smallest to 22 at the largest).

Using a template based on interview domains, the qualitative interview team (SES, AH and others) generated brief summaries of each interview transcript. The team lead (AH) then developed a matrix to examine content in each domain (x-axis) by participant (y-axis).^19^ Domains most relevant to EBQI-PACT implementation included VAIL awareness/familiarity, implementation of and participation in VAIL QI infrastructure (Quality Councils, Steering Committee, Workgroups), and experiences with local QI innovation projects at demonstration practices. To assess specific EBQI initiation and end dates, the core intervention team (SES, NS, LR) reviewed emails, meeting minutes, and quarterly reports.

Based on document review and matrix analysis, SES, NS, LR and AH developed a consensus rating for each logic model output (i.e., organizational structure or activity). Differences of opinion led to re-review of relevant data. Ratings were Met (implementation of this activity was complete at all demonstration sites), Partially Met (implementation was complete at some sites or partially complete at all sites), or Not Met (implementation was not complete at any site).

## RESULTS

### The EBQI-PACT Logic Model

**Figure 2:**
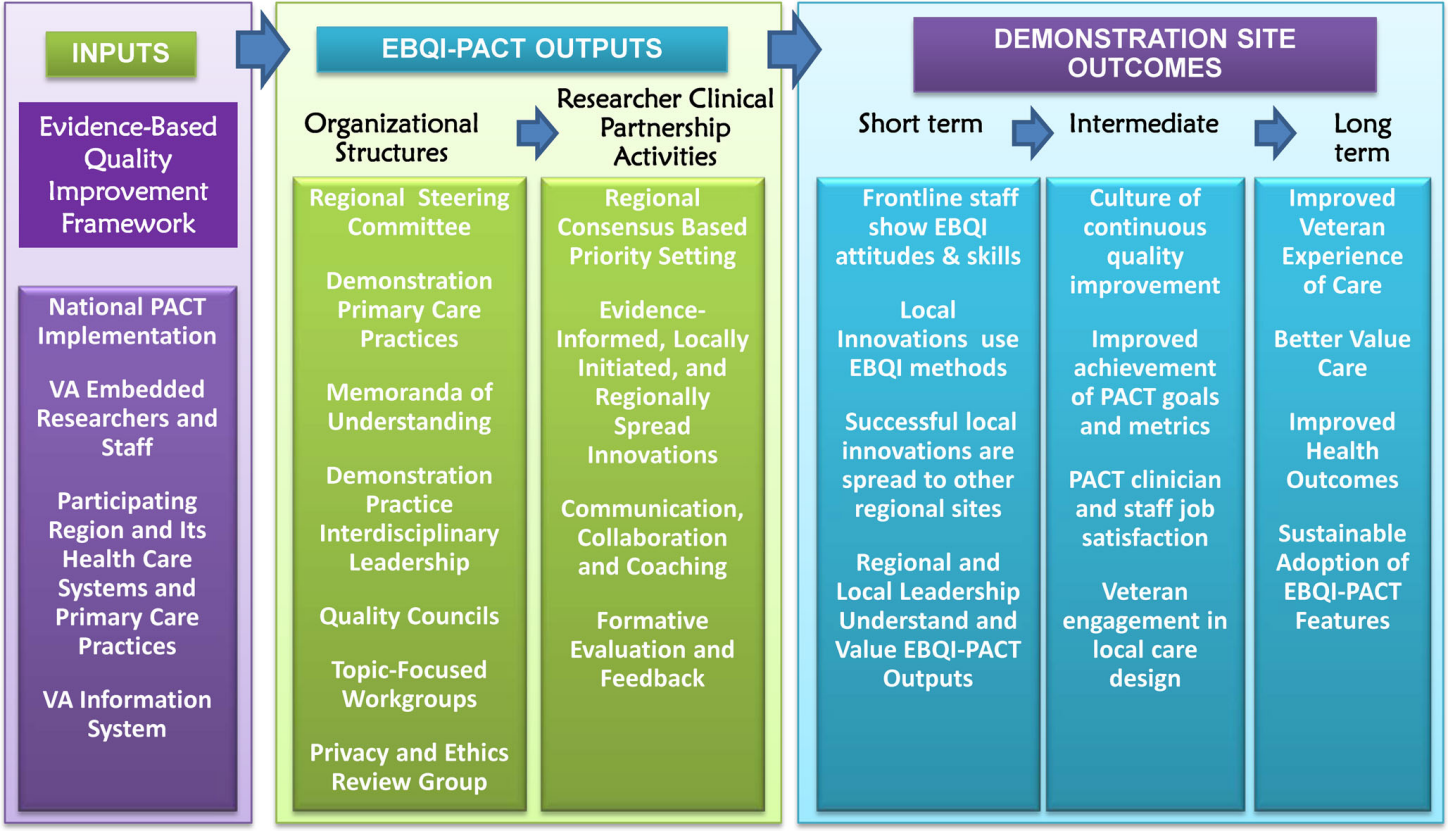
VAIL logic model for facilitating implementation of Patient Aligned Care Teams (PACT) using Evidence-Based Quality Improvement (EBQI) methods.

Figure 2 shows the final EBQI-PACT logic model.^20^ Inputs identify important pre-existing elements (people, framework, resources) that influence the conduct and outcomes of a project. Outputs identify what the intervention does to achieve its goals (i.e., program components implemented). Outcomes show what the outputs aim to achieve. Qualitative interviews identified barriers to implementation of logic model outputs, and are discussed below.

#### Logic Model Inputs

##### National PACT Implementation

VA used an extensive centrally designed package of directives, special-purpose funding, education, regional QI learning sessions, and PACT performance measures to implement PACT.^3^ The required staffing model focuses on interdisciplinary “teamlets”^21^^,^ 22 that follow patient panels in continuity. Teamlets are supported by a team that may include a health coach, social worker, and pharmacist, and a “neighborhood” of linked specialists and facilities.^23^

##### VA Embedded Researchers and Staff

The research team is based in a VA health services research center.

##### VA Information System

EBQI-PACT is occurring within the context of an integrated, highly developed electronic medical record and informatics infrastructure.^24^

#### Logic Model Outputs—Organizational Structures

##### Regional Steering Committee

Convened by VAIL, the Committee consists of regionally recognized leaders in information technology, patient advocacy, training, QI and redesign, and pharmacy. The Committee establishes innovation priorities.

##### Demonstration PC Practices

Demonstration practices are designated by their local HCS leaders as sites for developing, testing and spreading PACT-related innovations.

##### Healthcare System Memoranda of Understanding (MOU)

The top leadership (Director, Chief of Staff, Chief of Nursing) for participating HCSs agrees to support demonstration site Quality Councils, local interdisciplinary leadership, and release time for involved clinicians and staff.

##### Demonstration Practice Interdisciplinary Leadership

Each participating HCS identifies, for each demonstration practice, a local PC practice-based MD, nurse, and administrative leadership team.

##### Quality Councils

VAIL Quality Councils provide interdisciplinary QI leadership for each demonstration PC practice. They are charged with developing and/or reviewing all site QI innovations, selecting some to propose to the Steering Committee, engaging patient representatives, and meeting regularly. Each Council is supported by a Masters-level Coordinator hired by the local HCS, but supported through funds from VAIL. Phase 2 sites share a Coordinator with the Phase 1 site from their HCS (a planned total of three Coordinators).

##### Topic-Focused QI Workgroups

Five VAIL topic-focused Workgroups (education/training; mental health care in PACT; PACT for homeless; PACT pharmacy care; and patient-centered care) have across-site membership, engage patient representatives, meet regularly, and develop relevant innovations. Workgroups are led by regionally recognized experts, often with research experience. Members may be from outside VA.

##### Privacy and Ethics Review Subcommittee

Chaired by human subject research experts, this Subcommittee of the Steering Committee assists innovation projects.

#### Logic Model Outputs: Researcher-Clinical Partnership Activities

##### Regional Consensus-Based Priority Setting^4^^,^^15^

Once a year, Steering Committee members review submitted proposals using a structured online process, and come to consensus during a face-to-face meeting.

##### Evidence-Informed, Locally Initiated QI Innovations and Toolkits

Appendix 2 (available online) lists the innovations undertaken as of December 2012. Successful innovations produce toolkits addressing all relevant Chronic Care Model elements.^25^^,^ 26 Toolkits are pre-tested by additional demonstration sites prior to broader dissemination. Researchers provide technical support to innovations including: 1) responsive evidence review;^18^ 2) quality measures and information technology support; and 3) tool development and production.

##### Communication, Collaboration and Coaching

Activities include 1) twice-yearly collaborative learning sessions highlighting all innovations and their developers, with coaching for QI skill development;^27^ 2) twice monthly across-site coordination and learning calls; 3) participation in the regional PC committee; 4) biannual demonstration site newsletters; 5) VAIL website; and 6) coaching for Quality Council Coordinators, who in turn coach Quality Council members.

##### Formative Evaluation and Feedback

Focuses on user friendly formats showing PACT-relevant results across various data sources for demonstration sites.

#### Logic Model Outcomes

The logic model (Fig. 2) links features of the context (Inputs) and program components (Outputs) to their desired outcomes in the short-term, medium-term and long-term.

### Implementation Of EBQI-PACT

The analyses presented here address implementation of EBQI-PACT Outputs (Organizational Structures and Researcher Clinical Partnership Activities) at 18 months. Nearly all EBQI-PACT Outputs were in place or implemented, at least in part (Table 2). All sites remain engaged in EBQI as of October 2013.

**Table 2:** EBQI-PACT Implementation Results (June 2010 to December 2012)

EBQI-PACT outputs	Implementation activity	Achievement
Organizational structure outputs		
Regional Steering Committee	At least 80 % of Committee members	
	• Attend Steering Committee meetings	Met
	• Complete innovation priority reviews	Met
Demonstration primary care practices	100 % of participating healthcare systems	
	• Select three demonstration sites by 7/2010	Met
	• Select three additional sites by 9/2010	Met
Memoranda of Understanding (MOU) with healthcare systems	100 % of participating health systems meet MOU stipulations for	Met
	• Convening quality councils	Met
	• Naming site interdisciplinary leaders	Partially met
	• Release time for interdisciplinary leaders	Met
Demonstration practice interdisciplinary leadership	100 % of designated site leadership teams	
	• Include nurse, MD, and administrator	Met
	• Meet together regularly	Partially met
Quality councils	100 % of Sites Participate in Quality Councils that	
	• Have interdisciplinary membership (MD, RN, admin, pharmacy, social work)	Met
	• Include at least one patient representative	Partially met
	• Meet regularly	Partially met
	• Have a Quality Council Coordinator	Met
Topic focused workgroups	100 % of VAIL-convened Topic Focused Workgroups	
	• Have across site representation	Partially met
	• Include patient representatives	Partially met
	• An approved innovation project	Partially met
Privacy and ethics subcommittee	Privacy and Ethics Subcommittee convened and meets regularly for QI project review and ethical guidance.	Met
Researcher clinical partnership activities		
Regional consensus-based priority setting	5–8 innovations proposals are prioritized for VAIL support each year	Met
Evidence-Informed, locally initiated and regionally spread innovations	At least 80 % of VAIL-approved proposals have	
	• Requested and received an evidence review	Met
	• Use PDSA cycles (small tests of change)	Met
	At least 80 % of VAIL approved proposals have consulted with researcher technical support for production and development of	
	• Measures and/or information technology interventions	Met
	• Development of toolkits	Met
Communication, collaboration and coaching	100 % of demonstration sites participate at least 80 % of the time in	
	• Learning sessions (at least three representatives per site)	Met
	• Across site coordination and learning calls (at least one representative on each call)	Met
	• Local biannual newsletter production	Met
	100 % of Quality Council Coordinators attend at least one of the following researcher partnership activities at least 80 % of the time	
	• Coordinator coaching and leadership calls	Partially met
	• Measures and information technology support	Partially met
	• Learning sessions	Met
	The VAIL SharePoint site shows at least	
	• Ten non-researcher visitors/week	Met
	• Evidence of use of user feedback and response at least once per toolkit	Not met
Formative evaluation and feedback	At least once yearly, site level reports reflecting multiple data sources are shared with sites and regional leadership	Met

#### Implementation of EBQI-PACT Organizational Structure

As shown in Table 2, the *Regional Steering Committee* completed all implementation activities. Selection of *demonstration sites* was completed; however, one healthcare system had to identify a new site 6 months after start-up, due to a reorganization that eliminated the original site.

The implementation of EBQI-PACT interdisciplinary organizational structures varied somewhat across demonstration sites. The terms of the *MOU* for identifying local PC practice MD, RN and Administrative leaders were met in four of the six sites. *Quality Councils* in all six sites involved nursing, physicians, and administration in interdisciplinary leadership to some degree. All systems hired *Quality Council Coordinators*, and one hired two coordinators for a total of four positions. At four of six sites, Quality Councils met regularly and had substantial local authority over PACT QI activities. In two other sites, the Councils met intermittently except when their projects were in an active phase. Two HCSs created a new approach by linking their local VAIL Quality Councils to a larger system-level quality leadership group. Four Councils had patient representatives. All Quality Councils garnered one to three approved innovations.

Among the five *Topic-Focused Workgroups*, implementation activities were not completely met. One Workgroup included a patient representative, two had across-site representation, and three garnered an approved innovation. The *Privacy and Ethics Subcommittee* met expectations by reviewing all VAIL QI innovation projects to identify potential for unintended consequences, and providing consultation to two projects.

#### Qualitative Assessment of Barriers to Major EBQI-PACT Features (Outputs)

VA HCSs are organized in services (e.g. MD, nurse, administration). Local PC practice professionals and staff report upward to HCS service chiefs. Establishment of a named interdisciplinary leadership team and Quality Councils at local practices was a new activity for all demonstration sites. Four sites, however, had prior relevant local leadership experience and were more easily able to engage all or nearly all relevant leaders. Based on interview summaries, at the two sites not fully achieving Quality Councils, not all key interdisciplinary PC leaders joined the site’s Quality Council. One said: “There really needs to be better communication and coordination…But that may just be my lack of seeing what’s going on because I’m not on the [Quality Council], and that may just be the way it looks to me because I don’t know who’s doing what and how.” At these sites, some interviewees voiced frustration with leadership silos as an impediment to moving innovations beyond pilot phases.

The productivity of some Workgroups, and the extent to which they met expectations, varied over the period of observation. For example, one Workgroup has attracted substantial external funding in addition to VAIL innovations, has published several papers, and met all of our implementation criteria. Another Workgroup met early in the project, lapsed, and is now carrying out a partially externally funded innovation, but has not fully met any of our criteria. A third was active for several months and had a VISN-wide impact on development of PACT team member roles, but had no VAIL-approved innovation projects. The availability of Workgroup leaders with sufficient release time from research, clinical or administrative duties appears to be a determinant of model adherence.

#### Implementation of Researcher-Clinical Partnership Activities

The *Regional Steering Committee* completely implemented its research-clinical partnership activities. The Committee carried out two rounds of proposal review. Of 60 proposals submitted; the Committee approved 15 to receive VAIL support. Only one approved project was not completed. VAIL spent approximately $180,000 to support innovation budget requests, and received approval for all release time requested by innovation leaders. The *Evidence Review Group* completed 18 responsive evidence reviews and presented them at collaborative learning sessions. Three *innovation toolkits* have undergone across-site pre-testing, two of these are on the VAIL website for external spread, and five others are in pre-testing. For *formative evaluation*, VAIL produced two sets of site-level reports (twelve total) assessing provider, staff and patient experiences. Based on these reports, leaders at four of six demonstration practices undertook projects to address provider burnout and/or patient satisfaction.

Among intended research-clinical partnership activities related to *communication*, and *collaboration and coaching*, all activities related to learning sessions, calls, and local newsletters were met. Participation in biweekly site leader calls and collaborative learning sessions was high across sites and disciplines. Site participation in specific work on measures (at a weekly measures call), coaching and outreach, and innovations was more variable. One site’s Quality Council Coordinator participated irregularly in coaching, measures, or leadership calls. All six demonstration sites produced biannual newsletters (a total of 11 with one representing two sites); four of these highlighted patient representatives.

#### Qualitative Analysis of Barriers to Researcher-Clinical Partner Activities

These activities were perceived as generally supportive across sites. Based on interviews, the protected time for innovations provided by the HCSs through the MOU facilitated VAIL activities. Key stakeholders at all sites cited Quality Council Coordinators as indispensable for facilitating PACT QI. As one key stakeholder described, “Suddenly we were becoming very dependent on the [Coordinators]…[They] were even able to do much more complex mining of the data so that we were able to look at things that we never knew [we could get].” HCSs also noticed Coordinator skills. Based on project documents, two of six total Coordinators hired during the project were promoted to VISN or HCS leader support positions. Two others required VAIL leadership intervention because their HCSs overly accessed their time.

## DISCUSSION

Achieving PACT (or PCMH) goals requires a level of collaboration and integration within, across, and outside of PC practices that is transformational.^9^ Nutting and colleagues in the PCMH National Demonstration Project identified having an organizational culture that promotes organizational learning and development as a key facilitator for PCMH success.^2^ PC practice settings that promote ongoing quality improvement and innovation^1^ support organizational learning. EBQI-PACT is a multi-level quality improvement approach for supporting local, regional and national organizational learning through local quality improvement innovation that addresses PCMH and PACT goals.

Future summative evaluation will test whether EBQI-PACT achieves its intended outcomes. In the work presented here, we describe the key elements and logic underlying EBQI-PACT. We document that a large VA region, its HCSs, and its demonstration primary care practices implemented nearly all pre-planned EBQI-PACT structural changes and activities to a moderate or high level of fidelity over a two-and-a-half-year period. Finally, we identify barriers to some of the changes.

EBQI-PACT, like other multi-level regional initiatives carried out through practice-based research networks,^28^ may provide a feasible way to link QI experts, researchers and clinical stakeholders for advancing PCMH goals. If successful, the approach can be tested in additional non-VA and VA regions or networks as a method for achieving PCMH/PACT improvement.

To participate in EBQI-PACT, each demonstration site is required to have a named MD, nurse, and administrator leadership team, a local interdisciplinary Quality Council, and a Quality Council Coordinator. Qualitative interviews identified these structural changes, researcher team technical support, and the HCS sanctioned release time for designated local leaders as facilitators for PACT implementation.

Not all initial implementation activities were completed in all sites. Patient engagement in Quality Councils was enthusiastically adopted in four of six sites, but sustained in only one. Feedback from patient representatives indicates that the role requires initial training, as well as time commitments that may be difficult for volunteer representatives to support over time. Methods for supporting the critical role of patients in care design are urgently needed. Achieving local interdisciplinary leadership also appears to be easier in some sites than others, likely based on local site history and HCS support for local site interdisciplinary interaction and leadership. Further development of methods for overcoming historical silos is imperative for achieving PACT goals.

This project focused on reconfiguring rather than adding new resources for improvement. Project monetary resources support only Quality Council Coordinators and modest requested innovation resources; innovation team members use release time provided by their HCSs. Quality Council Coordinators are hired by HCSs and are not dissimilar to individuals hired for other QI work. The technical support team acts primarily at a distance from the PC sites, and consists of embedded health services researchers and staff.

The work presented here has limitations. First, it does not address EBQI-PACT effectiveness. Also, as shown in the logic model, the applicability of the approach to non-VA settings is context-dependent. In terms of applicability within VA, sites were chosen by HCSs and may not be representative. However, they did include both teaching and non-teaching HCS-based and community-based PC practices. Finally, while we used formal qualitative methods, we targeted our analyses to assess implementation only. Full qualitative analyses will be subsequently completed.

In summary, the information presented here shows that this EBQI-PACT demonstration achieved most of its implementation goals. Future evaluation will test the outcomes of this approach, and consider its applicability to other VA and non-VA care systems.

## Electronic supplementary material

Below is the link to the electronic supplementary material.

Appendix 1 (PDF 238 kb)

Appendix 2 (DOCX 33 kb)
