# Supplementary figures and images for: A Patient-Centered Primary Care Practice Approach Using Evidence-Based Quality Improvement: Rationale, Methods, and Early Assessment of Implementation
Source: J Gen Intern Med. 2014 Apr 9;29(Suppl 2):589–97. doi: 10.1007/s11606-013-2703-y (PMC4070240; doi:10.1007/s11606-013-2703-y)

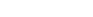

## VISN 22

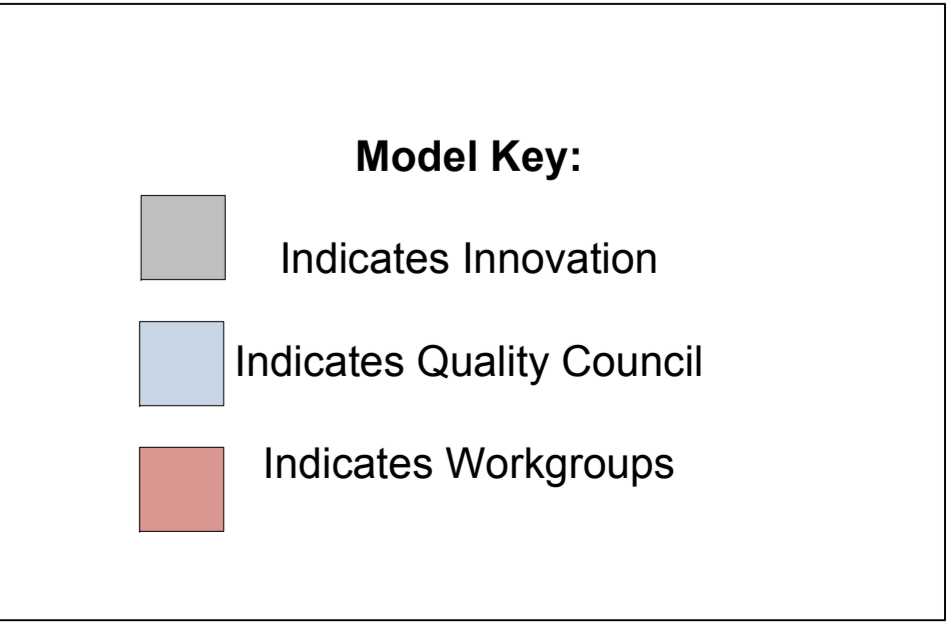

Supplement: Supplementary file 1 — (PDF 238 kb) [file 11606_2013_2703_MOESM1_ESM.pdf]
