# Supplementary material for: A Patient-Centered Primary Care Practice Approach Using Evidence-Based Quality Improvement: Rationale, Methods, and Early Assessment of Implementation
Source: J Gen Intern Med. 2014 Apr 9;29(Suppl 2):589–97. doi: 10.1007/s11606-013-2703-y (PMC4070240; doi:10.1007/s11606-013-2703-y)
Supplement: Supplementary file 2 — (DOCX 33 kb) [file 11606_2013_2703_MOESM2_ESM.docx]

**Appendix 2: Table of Phase I (2010-2011) & Phase II (2012) EBQI-PACT Innovation Projects**

| **Phase I Innovation Projects** | | |
| --- | --- | --- |
| **Innovation Title** | **Description** | **Status** |
| Point of Care Enrollment in MyHealtheVet Secure Messaging for Patients & Providers | Purpose: increase enrollment in MyHealtheVet by completing registration & authentication at the clinic visit. Project QI results for Version 1 of the toolkit showed an increase in authentication at the pilot clinic and a final comparison to the remaining medical center clinics of 19% versus 12%. Version 1 was spread and tested on one teamlet at another demonstration site proactive and the remaining teamlets at the medical center. Version 2 was tested across all three demonstration sites. | Project completed. Toolkit launched July 2012. |
| Local Provider Continuity Feedback | Purpose: enable teamlets to problem-solve around continuity issues through just-in-time every two week audit and feedback. Project QI results showed improved continuity from 50% to 75% at the pilot demonstration site per the PACT Compass over 12 months. The feedback provided to teamlets showed which patients experienced discontinuity over two weeks and why. This encouraged improvements such as changes in attending and trainee scheduling; local coding fixes; & links to the walk-in project by identifying discontinuity walk-ins. | Project completed. Toolkit launched April 2013. |
| Reducing Walk-in Visits for Pharmacy Refills | Purpose: reduce unnecessary walk-in visits to PACT for pharmacy refills. Project QI results showed decrease in average monthly walk-ins at pilot demonstration site of approximately 50% compared to “pre-PACT” (VistA data). Additional data collected showed reduction in walk-ins for refills from 50% to less than 33%. | Project completed. Toolkit in development. |
| PACT Post-Hospitalization Telephone Intervention | Purpose: determine the feasibility of conducting PACT provider/RN post-hospital discharge telephone interventions to Veterans within the current PACT structure. Project QI results from pilot demonstration site showed 83% of patients were reached by phone with completed encounters within 2 days of discharge. The median time spent on the phone was 15 mins. Completion of the innovation resulted in identification of issues related to nursing time allocation and to competing post-discharge call projects in V22. | Project completed. Toolkit in development. |
| TEX: Team Communication Exercise for PACT | Purpose: develop a coaching model and feedback tool for teamlets on developing healthy team relationships. Project QI results suggest that the acceptability of the intervention to participating team members is high. Although it was only tested with 2 teamlets so far, the TEX intervention shows promise for helping faculty and trainees to learn and improve the relational aspects of working in PACT teamlets and identifying key team communication behaviors such as active listening, consensus decision-making, constructive feedback, trust building, and tips for building relationships. | Project completed. Toolkit in development. |
| Mental Health Integration into PACT | Purpose: to improve coordination between primary care and mental health providers for continuity of care. Project QI results show an increase in the penetration (number of PC-MHI unique patients/total number of PC uniques) of PC-MHI at the pilot demonstration site from 5.6% in Fiscal Year 2010 to 7.3% in Fiscal Year 2011, reflecting an increase from 774 unique PC-MHI patients to 996 unique PC-MHI patients. The project led to a major redesign to achieve integrated care that is ongoing. | Project completed. Toolkit in development. |
| Care Coordination Home TeleHealth and Vet-to-Vet support for Homeless Veterans | Purpose: to improve the quality of care for veterans with a history of homelessness, by using Vet to Vet (V2V) peers to activate veterans in HUD-VA Supported Housing (HUD-VASH) to access PACT and Care Coordination Home TeleHealth (CCHT), a tele-medicine chronic disease management program. Project QI results from the pilot demonstration site showed that the 9 Veteran participants interviewed find the CCHT tele-buddy very easy to use. V2V peers help Veterans in HUD-VASH housing in numerous ways, including troubleshooting problems with the CCHT tele-buddy machine, providing information on how to contact their primary care provider, and enhancing connections to the VA. Potential barriers include recruitment and retention of trained V2V peer specialists. | Project completed. |
| RN Disease Manager Role in PACT | Purpose: to improve the level of wellness for patients with a chronic disease and empower a patient to become a consumer who participates in his/her care rather than a passive recipient of care. Project QI results at the pilot demonstration site showed that 75% of patients completed the group (6 of 8 patients). All 6 patients had successfully set goals and achieved their action plans by the last session. 3 patients experienced decreases in their Hemoglobin A1c’s , 2 experienced increases and patient 1 had an initial increase but expects it to decrease due to her now taking all meds as prescribed. 5 out of 6 patients (83%) knew their last Hemoglobin a1c’s and last blood sugar readings at the onset of the program and 5 out of 6 patients (83%) knew their last reading at the end of the program. These results suggest that participation in the diabetes group resulted in increased knowledge of diabetes care and patient goal-setting; however, high-risk patients with poorly controlled diabetes (A1c>9) may not be the best target for a group intervention. . | Project completed. |
| A Quality Improvement Approach to Communication between Primary Care & Mental Health Providers | Purpose: (1) Utilize continuous quality improvement (QI) tools (i.e., flow chart, fishbone diagram) to describe and analyze the process of communication between primary care providers and mental health providers for shared patients. (2) To develop an intervention(s) to facilitate communication between primary care providers and mental health providers for shared patients | Evaluation in progress & toolkit in development. |
| **Phase II Innovation Projects** | | |
| **Innovation Title** | **Description** | **Status** |
| Use of Super Huddles to improve care of high-risk patients | Purpose: to improve the care we provide our high-risk patients through the use of Super Huddles. Deliverables include: (1) a user-friendly tool for the teamlet to proactively identify high-risk patients (2) a tool to be used during and in-between Super Huddle meetings to allow tracking of actionable items, consults, etc., (3) process for convening interdisciplinary team meetings to coordinate care for high-risk patients; (4) measures for assessing success of the tools. | Project completed. Evaluation in progress and toolkit in development. |
| Nursing Post Discharge Note | Purpose: create a standardized process and template tool to capture post discharge follow up in order to increase care coordination with patients, prevent readmission and provide feedback information to the inpatient service about the effectiveness of the discharge instruction process. Deliverables include: 1. A comprehensive tool for spread; 2. Local measures for QI for post-hospital follow-up. | Project completed. Evaluation in progress and toolkit in development. |
| Improving Medication Renewal Efficiency | Purpose: to increase the number of renewals through the AudioRENEWAL System, reducing time demands on PACT teamlets and allowing for convenient, any-time first call resolution for patients requiring medication renewals. Expected deliverables: A tool identifying a process for increasing use of AudioCare to renew medications that would include protocol for setting up clinics, provider and patient education, and partnering with pharmacy to increase the number of medications eligible for refill through AudioRENEWAL process. | Project almost completed; reviewing data to determine impact |
| Spreading the PACT Emergency Department (ED) Follow-up Communication Tool | Purpose: (1) to spread to the PACT teams the specific/urgent follow-up communication tool and processes developed and (2) assess the effect of the implementation of the tool and processes on ED re-visits and post-ED unscheduled appointments. Expected deliverables: (1) A comprehensive tool for spread; (2) Local QI measures of ED/primary care communication and (3) A publication evaluating tool performance. | Project not completed – innovation is underway and needs further work |
| Screening for Homelessness: Feasibility and Implementation of a Tool to Identify Veterans who are Homeless and At-risk for Homelessness in ED and PACT Settings | Purpose: identify and implement a brief staff-administered screening tool (paper and pencil) to identify patients who are homeless or at-risk for homelessness that can be delivered at the point of care. Expected deliverables: (1) A comprehensive tool for spread and (2) A publication evaluating tool performance. A background paper on V22 resources for homelessness is in the revise and submit process. A second background paper on homelessness care data for the demonstration site is has been submitted for review. A third paper on engaging veteran support for improving health care for HUD-VASH veterans is in draft. | Project not completed – innovation is underway and needs further work |
| Introduction to Relaxation and Meditation | Purpose: provide an introduction to evidence-based, holistic, adjunctive relaxation & meditation treatments for primary care patients with emotional and health behavior concerns. Expected deliverables: (1) A toolkit with curricula and process for conducting a 4-week group visit for Introduction to Relaxation and Meditation. (2) Pre-post patient self-report measures of therapy group success. (3) A manuscript evaluating the results of the tool in one site. | Project not completed – innovation is underway and needs further work |
| Educating Medical Residents in Primary Care Mental Health Integration | Purpose: reorganize the educational approach to training medical residents in basic management of mental health concerns in primary care. Expected Deliverables: (1) A tool for educating medical residents about MH; (2) A set of tools to promote bidirectional communication between PC & MHSs; (3) Measures reflecting process and outcomes of PC/MHS communication; (4) A manuscript is in draft on Phase 1 needs assessment findings; a second manuscript is in preparation on PC provider knowledge and attitudes toward depression care; and additional manuscripts are planned. | Project not completed – innovation is underway and needs further work |
